# Immuno-related polymorphisms and cervical cancer risk: The IARC multicentric case-control study

**DOI:** 10.1371/journal.pone.0177775

**Published:** 2017-05-15

**Authors:** James McKay, Vanessa Tenet, Silvia Franceschi, Amélie Chabrier, Tarik Gheit, Valérie Gaborieau, Sandrine Chopin, Patrice H. Avogbe, Massimo Tommasino, Michelle Ainouze, Uzma Hasan, Salvatore Vaccarella

**Affiliations:** 1 International Agency for Research on Cancer, Lyon, France; 2 Centre International de Recherche en Infectiologie, International Center for Infectiology Research (CIRI), Lyon, France; 3 Inserm, U1111, Lyon, France; 4 Ecole Normale Supérieure de Lyon, Lyon, France; 5 University Lyon 1, Lyon, France; 6 CNRS, UMR5308, Lyon, France; 7 Laboratoire d'Immunologie, Hospices Civils de Lyon, Centre Hospitalier Lyon Sud, Lyon, France; Universidade Estadual de Maringa, BRAZIL

## Abstract

A small proportion of women who are exposed to infection with human-papillomavirus (HPV) develop cervical cancer (CC). Genetic factors may affect the risk of progression from HPV infection to cervical precancer and cancer. We used samples from the International Agency for Research on Cancer (IARC) multicentric case-control study to evaluate the association of selected genetic variants with CC. Overall, 790 CC cases and 717 controls from Algeria, Morocco, India and Thailand were included. Cervical exfoliated cells were obtained from control women and cervical exfoliated cells or biopsy specimens from cases. HPV-positivity was determined using a general primer GP5+/6+ mediated PCR. Unconditional logistic regression was used to estimate odds ratios (OR) and corresponding 95% confidence intervals (CI) of host genotypes with CC risk, using the homozygous wild type genotype as the referent category and adjusting by age and study centre. The association of polymorphisms with the risk of high-risk HPV-positivity among controls was also evaluated. A statistically significant association was observed between single nucleotide polymorphism (SNP) CHR6 rs2844511 and CC risk: the OR for carriers of the GA or GG genotypes was 0.70 (95% CI: 0.43–1.14) and 0.61 (95% CI: 0.38–0.98), respectively, relative to carriers of AA genotype (p-value for trend 0.03). We also observed associations of borderline significance with the TIPARP rs2665390 polymorphism, which was previously found to be associated with ovarian and breast cancer, and with the *EXOC1* rs13117307 polymorphism, which has been linked to cervical cancer in a large study in a Chinese population. We confirmed the association between CC and the rs2844511 polymorphism previously identified in a GWAS study in a Swedish population. The major histocompatibility region of chromosome 6, or perhaps other SNPs in linkage disequilibrium, may be involved in CC onset.

## Introduction

Although the burden of cervical cancer (CC) has decreased considerably over recent decades in countries that have implemented high-quality cytology screening programmes [1, 2], it remains the fourth most commonly diagnosed cancer worldwide among women and the most common site in several low-income countries [3]. Furthermore, incidence rates of CC are also expected to increase in the next decades in some areas of the world, e.g., Eastern European countries [4]. Persistent infection with high-risk human papillomavirus (HR-HPV) types is a necessary cause of CC. Genital infection with HPV is very common among the general female population, but only a small fraction of women develop a persistent infection [5] and are subsequently at risk for progression to precancer and invasive CC. Thus, other factors in addition to HPV are likely to be involved in progression from an infected cell to a transformed cell with invasive potential. Although there is a relatively large amount of information on the role of non-genetic co-factors [6–9], only a few studies, mainly targeting Caucasian or Chinese populations, have explored the association between host genetics, in particular host immune molecules, and the pathogenesis of CC [10–12].

The host immune response is involved in the persistence of HPV infection in certain individuals. For instance, Toll-like receptor 9 (TLR-9) is an essential component of innate immunity able to recognize double-stranded DNA molecules of viral origin and to elicit the production of immunostimulatory and pro-inflammatory cytokines including Type I interferon (IFN). Studies demonstrated that HPV16, the most oncogenic HPV type [13], interferes with the activity of TLR9 by decreasing its expression [14–19]. As virtually all CCs are caused by persistent HPV infection, women who inherit alleles that may affect the expression of molecules capable of recognising HPV infection might be at an increased risk of developing CC.

The main objective of the present study is to replicate the associations of genetic variants identified by a genome-wide association study (GWAS) [10–12] in previously unstudied populations. We used samples from the International Agency for Research on Cancer (IARC) multicentric case-control (IMCC) study on CC, including populations from Algeria, Morocco, India and Thailand. In addition, we evaluated the association of other polymorphisms in innate immune molecules with CC risk. After a systematic review of the literature, we selected a number of single nucleotide polymorphisms (SNP) based on one of the following criteria: 1) previously observed associations with cervical precancer/cancer or with HPV persistence [20]; 2) previously observed associations identified by GWAS with cancers other than CC; 3) demonstrated functional impairment by HPV infection [14–16].

## Materials and methods

### Study population

In the present analysis, we included four (of the nine) countries included in the IMCC: Algeria [21], Morocco [22], India [23] and Thailand [24]. Methods have been described in detail in the individual publications. Briefly, eligible cases were residents in predefined study areas, or women attending reference hospitals with incident, histologically confirmed invasive CC. A total of 1,013 cases were identified, mainly squamous-cell carcinomas but also including 84 adeno- or adenosquamous invasive cervical carcinomas. Control women were in-patients or out-patients from the same or other hospitals in the areas where cancer cases were recruited. Control women were frequency matched to cases by 5-year age group and did not include women admitted to hospital for cancers of the anogenital tract, breast and colon, smoking-related diseases, or sexually transmitted infections. A total of 973 control women were identified for the present analysis.

Informed written consent was obtained from all participants. The studies were approved by the IARC Ethical Review Committee and by local ethical committees in each of the participating countries (Ethics Committee of the Institut National d’Oncologie, Rabat, Morocco; Ethics review committee of the Centre Pierre et Marie Curie in Algiers, Algeria; Ethics review committee of the Cancer Institute in Chennai, India; Ethics Committee of the Cancer Centre in Hat-Yai, Thailand).

### Laboratory methods

Study samples for DNA extraction included buffy coats, paraffin-embedded tumour biopsies, cervical cells in liquid-based cytology media or cervical cells in phosphate-buffered saline and were available for 912 cases and 822 controls. Specific DNA extraction protocols were envisaged for different types of samples.

### DNA extraction for host genetic analyses

In Algeria and India, DNA was extracted from buffy coats using the EZ1 DNA blood 350 μl Kit (Qiagen, Hilden, Germany) according to the manufacturer’s protocol. A total of 195 cases and 187 controls in Algeria, and 132 cases and 174 controls in India had good quality DNA and were included in the present analysis.

In Morocco and Thailand, DNA was extracted from cervical cells using the QIAamp DNA mini kit (Qiagen Hilden, Germany). For paraffin-embedded tumour biopsies, the DNA was extracted as previously described [25]. Overall, a total of 183 cases and 145 controls in Morocco and 280 cases and 211 controls in Thailand had good quality DNA and were also included.

### Host genotyping

Genotyping of genetic variants was performed using the TaqMan genotyping platform (Lifetechnologies), as described previously [26]. Taqman probes could not be designed for genotypes rs25164488, rs3117027 and rs9272143, hence we used three variants in high linkage disequilibrium (LD), rs2844511, rs3129269 and rs9272105, respectively, as proxy markers. The robustness of the Taqman assays was confirmed at IARC by re-genotyping the CEPH HapMap (CEU) trios and confirming concordance with genotypes derived from the Hapmap project (http://hapmap.ncbi.nlm.nih.gov/cgi-perl/gbrowse/hapmap24_B36/). Robust separation of genotype clusters was impossible only for rs9272105 and hence this variant was not included in the present report. The genotype distributions (in controls and by country of origin) were in line with that expected assuming Hardy Weinberg Equilibrium.

### HPV testing

HPV testing was performed in the Department of Pathology at the Vrije University Medical Center, Amsterdam, the Netherlands, as described in the individual study publications [27, 28]. The overall presence of HPV DNA in cervical exfoliated cells from control subjects and cervical exfoliated cells or biopsy specimens from case subjects was determined by performing a general primer GP5+/6+ mediated PCR [29]. HPV positivity was assessed by hybridisation of PCR products in an enzyme immunoassay using two HPV oligoprobe cocktails that, together, detect the following HPV types: HPV6, 11, 16, 18, 26, 31, 33, 34, 35, 39, 40, 42, 43, 44, 45, 51, 52, 53, 54, 55, 56, 57, 58, 59, 61, 66, 68, 70, 72, 73, 82 (IS39 and MM4 subtypes), 83 (equivalent to MM7), 84 (equivalent to MM8), and CP6108. Subsequent HPV typing was performed by reverse-line blot hybridisation of PCR products, [27, 28]. HPV16, 18, 31, 33, 35, 39, 45, 51, 52, 56, 58, 59 and 68 were considered HR types in the present report.

### Statistical analyses

Unconditional logistic regression was used to estimate odds ratios (OR) and corresponding 95% confidence intervals (95% CI) for the association of each genotype with CC risk, using the homozygous wild type (WT) genotype as the referent group. The analyses were adjusted for age (<40, 40–49, 50–59, ≥60) and study centre. The association between each genetic variant and CC risk was also estimated assuming a log additive genetic model. Tests for trend were computed using the three-level variables (0, 1, 2) of homozygote wildtype, heterozygote, and homozygote variant as continuous variables. When small cell sizes were found (less than 5%), the combined effect of homozygote and heterozygote variants versus wildtype was assessed. Heterogeneity of ORs between countries was tested by fitting separate models to each area and then comparing the observed with the expected dispersion of estimates around the pooled mean using a chi-squared statistic. We also evaluated the association between SNPs and HR-HPV positivity among controls. In this case, the focus is not on cancer risk, but rather on the risk for healthy women to be infected with HR-HPV types. We therefore considered HR-HPV-positivity among controls as the outcome (dependent variable) and evaluated the excess (or deficit) in the risk of being HR-HPV-positive, as compared to being HR-HPV-negative, in relation to carrying a particular allele of the SNP. The association between SNPs and positivity for any HPV type among controls was used to assess sensitivity. Analyses were conducted using R and SAS version 9.4.

## Results

A total of 790 women diagnosed with CC and 717 controls were retained for the present analysis (Table 1). Mean age was 50.8 (ranging from 47.8 in India to 54.4 in Algeria) for cases and 48.3 (ranging from 40.6 in Morocco to 52.8 in Algeria) for controls. Overall, 730 cases had valid HPV results, and among these 688 (94.3%) were positive for HR-HPV infection. Among control women, 642 had valid HPV results, of which 74 (11.5%) were positive for HR-HPV infection. Table 2 shows the results of the association of 21 selected SNPs with CC risk. This analysis identified one SNP to be statistically significantly associated with CC at a p-value of 0.05, chr6-MHC rs2844511 (p-value 0.03). Carriers of the GA or GG genotypes for the SNP CHR6 rs2844511 demonstrated a 30% and 39% reduction in disease risk respectively, relative to carriers of AA genotype. For two other SNPs, TIPARP rs2665390 and *EXOC1* rs13117307, we found associations of borderline statistical significance (p-values 0.05 and 0.06, respectively), with TC carriers of TIPARP rs2665390 showing an increased risk of CC (OR_TC_ = 1.41, 95% CI: 1.00–1.99) as compared to TT carriers, and with CT and TT carriers in *EXOC1* rs13117307 showing a reduced risk (OR_CT_ = 0.81, 95% CI: 0.63–1.03; OR_TT_ = 0.72, 95% CI: 0.36–1.45).

**Table 1:** Characteristics of subjects included in the analyses by study.

IARC case-control studies				
	Cases		Controls	
	n	(%)	n	(%)
**Total in original studies**	1013		973	
**Valid genotyping results**	790		717	
**Study**				
Morocco	183	(23.2)	145	(20.2)
Algeria	195	(24.7)	187	(26.1)
Thailand	280	(35.4)	211	(29.4)
India	132	(16.7)	174	(24.3)
**Age group**				
<40	138	(17.5)	174	(24.3)
40–49	232	(29.4)	209	(29.2)
50–59	214	(27.1)	192	(26.8)
≥60	206	(26.1)	142	(19.8)

**Table 2:** Odds ratios* and 95% confidence intervals for candidate SNPs for associations with invasive CC, among 790 cases and 717 controls. The IARC multicentric CC case-control study.

Gene	SNP	Genotype	Cancer cases		Controls		OR (95% CI)
			N	%	N	%	
*CHR6*	RS2844511	AA	51	6.7	31	4.5	1.00 (ref)
		GA	272	35.8	235	34.2	0.70 (0.43–1.14)
		GG	437	57.5	422	61.3	**0.61 (0.38–0.98)**
		Log-add					**OR = 0.83 (0.70–0.99); p-trend = 0.03**
*CHR6*	RS3129269	AA	156	21.6	133	20.1	1.00 (ref)
		AC	317	44.0	329	49.6	0.79 (0.60–1.05)
		CC	248	34.4	201	30.3	0.98 (0.73–1.33)
		Log-add					OR = 1.02 (0.87–1.18); p-trend = 0.85
*EXOC1*	RS13117307	CC	559	74.2	470	69.0	1.00 (ref)
		CT	179	23.8	191	28.1	0.81 (0.63–1.03)
		TT	15	2.0	20	2.9	0.72 (0.36–1.45)
		Log-add					**OR = 0.82 (0.67–1.01); p-trend** = **0.06**
*CHR17*	RS8067378	AA	287	39.0	275	41.8	1.00 (ref)
		AG	322	43.8	279	42.4	1.13 (0.89–1.43)
		GG	127	17.3	104	15.8	1.17 (0.84–1.61)
		Log-add					OR = 1.09 (0.93–1.27); p-trend = 0.28
*SLIT3*	RS11134527	GG	285	37.6	247	36.1	1.00 (ref)
		GA	323	42.6	297	43.4	0.93 (0.73–1.19)
		AA	151	19.9	141	20.6	0.91 (0.67–1.25)
		Log-add					OR = 0.95 (0.82–1.11); p-trend = 0.54
*CHR10*	RS10886416	CC	550	71.8	513	73.7	1.00 (ref)
		CA	195	25.5	163	23.4	1.18 (0.92–1.53)
		AA	21	2.7	20	2.9	1.00 (0.53–1.91)
		Log-add					OR = 1.14 (0.90–1.38); p-trend = 0.32
C17ORF99	RS9893818	CC	750	98.4	681	98.4	1.00 (ref)
		CA	11	1.4	11	1.6	0.90 (0.38–2.13)
		AA	1	0.1	0	0.0	
*DMRTC2*	RS2305809	TT	217	28.5	182	26.1	1.00 (ref)
		TC	368	48.3	351	50.4	0.83 (0.65–1.06)
		CC	177	23.2	164	23.5	0.89 (0.67–1.20)
		Log-add					OR = 0.94 (0.81–1.09); p-trend = 0.41
IL10RA	RS9610	AA	229	30.7	221	32.0	1.00 (ref)
		AG	357	47.9	339	49.1	1.03 (0.81–1.31)
		GG	160	21.5	130	18.8	1.20 (0.89–1.63)
		Log-add					OR = 1.09 (0.94–1.26); p-trend = 0.26
*IL12RB2*	RS4297265	GG	305	40.2	251	36.4	1.00 (ref)
		GA	337	44.5	332	48.1	0.87 (0.69–1.10)
		AA	116	15.3	107	15.5	1.01 (0.73–1.39)
		Log-add					OR = 0.97 (0.84–1.13); p-trend = 0.73
*IL12RB2*	RS2229546	AA	205	27.2	205	30.1	1.00 (ref)
		AC	361	47.9	323	47.2	1.06 (0.83–1.36)
		CC	188	24.9	154	22.6	1.10 (0.81–1.48)
		Log-add					OR = 1.05 (0.90–1.22); p-trend = 0.54
*TLR9*	RS5743836	AA	632	81.8	569	81.6	1.00 (ref)
		AG	129	16.7	113	16.2	1.10 (0.82–1.47)
		GG	12	1.6	15	2.2	0.73 (0.33–1.58)
		Log-add					OR = 1.01 (0.79–1.28); p-trend = 0.96
*TLR9*	RS352140	CC	308	40.8	265	38.4	1.00 (ref)
		CT	323	42.8	312	45.2	0.89 (0.71–1.12)
		TT	124	16.4	114	16.5	0.94 (0.69–1.28)
		Log-add					OR = 0.95 (0.82–1.11); p-trend = 0.53
*HPV8*	RS114427648	AA	717	93.0	646	92.4	1.00 (ref)
		AG	53	6.9	53	7.6	0.92 (0.61–1.39)
		GG	1	0.1	0	0.0	
*TERT*	RS2736100	CC	275	38.4	249	36.9	1.00 (ref)
		CA	301	42.0	305	45.2	0.86 (0.68–1.09)
		AA	140	19.6	121	17.9	0.96 (0.71–1.31)
		Log-add					OR = 0.96 (0.83–1.11); p-trend = 0.59
*CLTMP1L*	RS402710	GG	342	45.2	317	46.3	1.00 (ref)
		GA	309	40.8	276	40.3	0.97 (0.77–1.22)
		AA	106	14.0	92	13.4	0.97 (0.69–1.36)
		Log-add					OR = 0.98 (0.83–1.15); p-trend = 0.81
*CASP8*	RS3769825	AA	348	46.2	314	45.3	1.00 (ref)
		AG	305	40.5	288	41.6	0.94 (0.75–1.19)
		GG	101	13.4	91	13.1	1.00 (0.71–1.41)
		Log-add					OR = 0.98 (0.84–1.16); p-trend = 0.86
*TIPARP*	RS2665390	TT	677	87.5	626	90.3	1.00 (ref)
		TC	93	12.0	65	9.4	**1.41 (1.00–1.99)**
		CC	4	0.5	2	0.3	-
*TIPARP*	RS7651446	GG	740	95.9	678	97.1	1.00 (ref)
		GT	29	3.8	20	2.9	1.30 (0.72–2.35)
		TT	3	0.4	0	0.0	-
*PRKAA*	RS13361707	CC	295	38.7	263	38.0	1.00 (ref)
		TC	337	44.2	320	46.2	0.88 (0.71–1.11)
		TT	130	17.1	110	15.9	0.97 (0.71–1.33)
		Log-add					OR = 0.97 (0.83–1.12); p-trend = 0.64
*ZBTB20*	RS9841504	CC	586	76.3	517	74.7	1.0 (ref)
		CG	172	22.4	158	22.8	0.99 (0.77–1.28)
		GG	10	1.3	17	2.5	0.61 (0.27–1.36)
		Log-add					OR = 0.93 (0.74–1.16); p-trend = 0.51

* OR adjusted for centre and age

**Fig 1:**
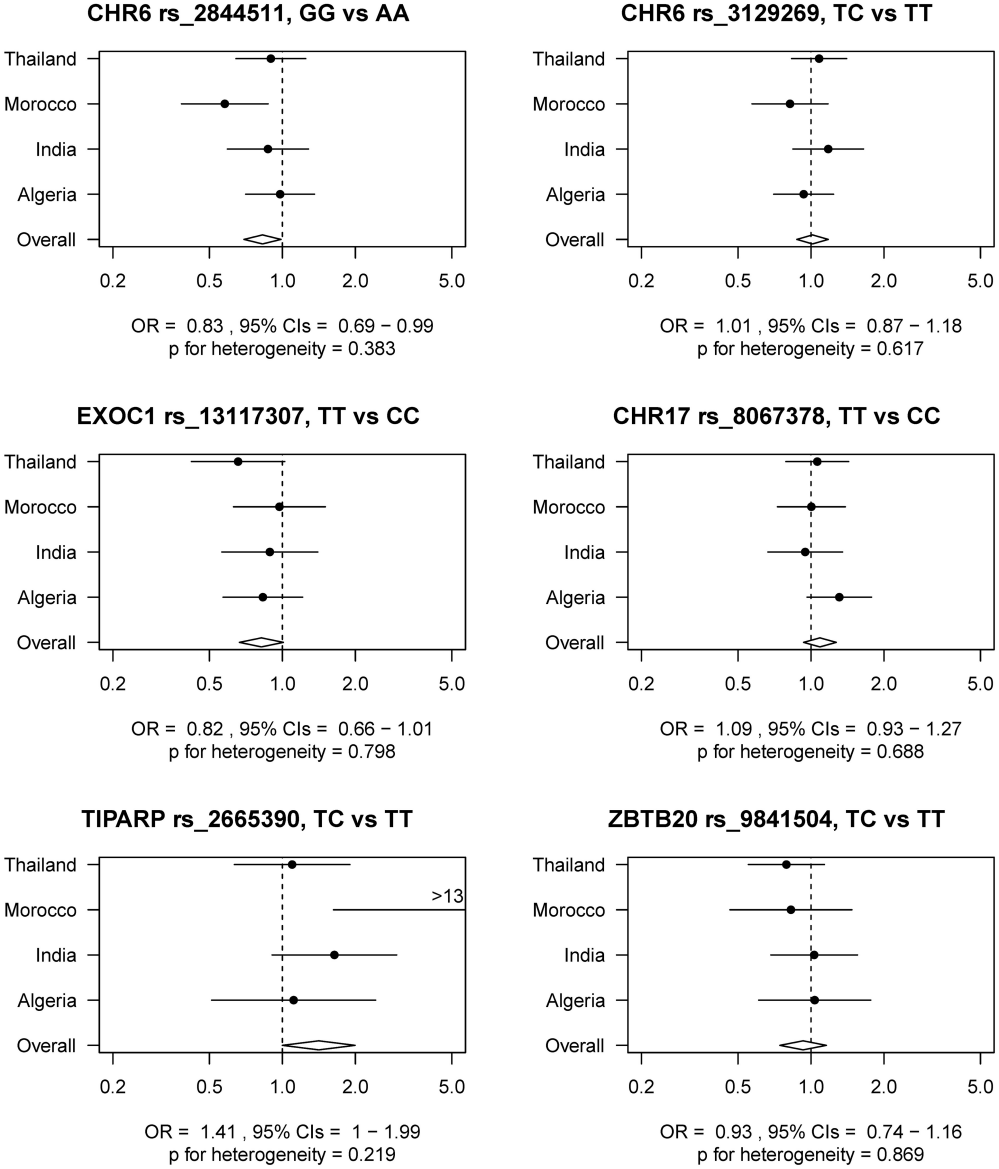
Association between selected SNPs and risk of cervical cancer, overall and by country.

Fig 1 shows the association of six selected SNPs by country with CC, according to the log additive genetic model. Besides the three SNPs that showed a significant or borderline significant association with CC, we also included results for the three other SNPs, CHR6 rs3129269, EXOC1 rs8067307, and ZBTB20 rs9841504. There was some variation in the associations between countries although none demonstrated significant evidence for heterogeneity.

Table 3 shows the association of the studied SNPs with the presence of HR-HPV infection among controls only. A statistically significant association was observed for rs13117307 in *EXOC1*, with individuals with a genotype of TT for the SNP *EXOC1* rs13117307 having a 3-fold increase (95% CI: 1.09–10.6) in risk of being HR-HPV-positive as compared to individuals with a genotype CC, p-value for trend 0.04. AA carriers of *TERT* rs2736100 had a significantly reduced risk (OR = 0.27, 95% CI: 0.09–0.81) of HR-HPV-positivity as compared to CC carriers, although the p-value for the trend was of a borderline statistical significance (0.05).

**Table 3:** Odds ratios* and 95% confidence intervals for the associations of candidate SNPs with HR- HPV infection, among 717 controls. The IARC multicentric CC case-control study.

Gene	SNP	Genotype	Among controls: HR-HPV+ vs HR-HPV- OR (95% CI)
*CHR6*	RS2844511	AA	1.00 (ref)
		GA	0.68 (0.18–2.59)
		GG	1.04 (0.29–3.78)
		Log-add	OR = 1.13 (0.81–2.12); p-trend = 0.27
*CHR6*	RS3129269	AA	1.00 (ref)
		AC	1.24 (0.60–2.58)
		CC	1.85 (0.85–4.04)
		Log-add	OR = 1.38 (0.94–2.03); p-trend = 0.10
*EXOC1*	RS13117307	CC	1.0 (ref)
		CT	1.38 (0.78–2.45)
		TT	**3.39 (1.09–10.58)**
		Log-add	**OR = 1.59 (1.02–2.48);** p-trend = **0.04**
*CHR17*	RS8067378	AA	1.0 (ref)
		AG	0.63 (0.35–1.14)
		GG	0.99 (0.48–2.07)
		Log-add	OR = 0.91 (0.63–1.32); p-trend = 0.62
*SLIT3*	RS11134527	GG	1.00 (ref)
		GA	0.91 (0.51–1.64)
		AA	0.72 (0.33–1.55)
		Log-add	OR = 0.85 (0.59–1.24); p-trend = 0.41
*CHR10*	RS10886416	CC	1.0 (ref)
		CA	1.14 (0.60–2.05)
		AA	0.99 (0.21–4.61)
		Log-add	OR = 1.07 (0.64–1.76); p-trend = 0.80
C17ORF99	RS9893818	CC	1.00 (ref)
		CA	2.60 (0.52–13.12)
		AA	
*DMRTC2*	RS2305809	TT	1.00 (ref)
		TC	1.02 (0.56–1.87)
		CC	1.04 (0.50–2.13)
		Log-add	OR = 1.02 (0.71–1.46); p-trend = 0.92
IL10RA	RS9610	AA	1.00 (ref)
		AG	1.71 (0.92–3.15)
		GG	1.38 (0.64–2.97)
		Log-add	OR = 1.20 (0.84–1.72); p-trend = 0.31
*IL12RB2*	RS4297265	GG	1.0 (ref)
		GA	0.88 (0.50–1.53)
		AA	0.64 (0.29–1.43)
		Log-add	OR = 0.82 (0.56–1.19); p-trend = 0.29
*IL12RB2*	RS2229546	AA	1.0 (ref)
		AC	0.99 (0.53–1.84)
		CC	1.77 (0.87–3.60)
		Log-add	OR = 1.32 (0.91–1.91); p-trend = 0.15
*TLR9*	RS5743836	AA	1.0 (ref)
		AG	0.92 (0.45–1.86)
		GG	1.82 (0.38–8.66)
		Log-add	OR = 1.07 (0.61–1.88); p-trend = 0.82
*TLR9*	RS352140	CC	1.0 (ref)
		CT	1.09 (0.63–1.89)
		TT	0.84 (0.39–1.83)
		Log-add	OR = 0.95 (0.67–1.36); p-trend = 0.80
*HPV8*	RS114427648	AA	1.0 (ref)
		AG	0.18 (0.02–1.33)
		GG	
*TERT*	RS2736100	CC	1.00 (ref)
		CA	1.05 (0.61–1.82)
		AA	**0.27 (0.09–0.81)**
		Log-add	**OR = 0.69 (0.47–1.01); p-trend = 0.05**
*CLTMP1L*	RS402710	GG	1.00 (ref)
		GA	0.79 (0.43–1.42)
		AA	1.10 (0.46–2.66)
		Log-add	OR = 0.96 (0.63–1.46); p-trend = 0.85
*CASP8*	RS3769825	AA	1.0 (ref)
		AG	0.54 (0.30–0.98)
		GG	0.90 (0.42–1.93)
		Log-add	OR = 0.82 (0.56–1.21); p-trend = 0.33
*TIPARP*	RS2665390	TT	1.00 (ref)
		TC	1.10 (0.48–2.55)
		CC	
*TIPARP*	RS7651446	GG	1.00 (ref)
		GT	0.56 (0.07–4.40)
		TT	
*PRKAA*	RS13361707	CC	1.0 (ref)
		TC	1.36 (0.77–2.40)
		TT	1.25 (0.56–2.79)
		Log-add	OR = 1.17 (0.81–1.69); p-trend = 0.42
*ZBTB20*	RS9841504	CC	1.0 (ref)
		CG	0.98 (0.54–1.78)
		GG	1.46 (0.38–5.63)
		Log-add	OR = 1.07 (0.66–1.73); p-trend = 0.80

* OR adjusted for centre and age

## Discussion

In this international multicentric case-control study of CC that included women from Morocco, Algeria, India and Thailand, we confirmed the association of the rs2844511 polymorphism previously identified in a GWAS study in a Sweden population [10] with CC.

We showed that the distribution of alleles was significantly different between cases and controls for the rs2844511 polymorphism. This observation is consistent with and of similar magnitude to the reduction in risk for rs2844511 GG as compared to AA carriers previously observed by a GWAS study in a Swedish population [10], (OR in Chen et al. = 0.70, 95% CI: 0.63–0.77). Interestingly, the reference category in Chen et al. [10] was set to be the AA carriers as this combination of alleles was the most frequent in that Swedish study. In our international study population, however, the AA allele combination was relatively rare in controls (between 3–6%, depending on the country), an observation consistent with the large variations in allele frequencies observed within this dynamic part of the human genome. Nevertheless, for consistency and comparability with [10], we have maintained AA as reference category.

The rs2844511 SNP is a proxy for SNP rs2516448, which was also previously linked to CC [10] with the minor allele being associated with an increased risk, and is located on the *MICA* gene within the major histocompatibility complex (MHC) region at 6p21.3. The MICA protein is a stress-induced highly polymorphic, epithelia-specific membrane-bound glycoprotein interacting with the activating NK cell receptor NKG2D [30]. Importantly, a previous study has reported that MICA protein is absent from most cells, but can be induced by viral infection, with its expression being frequent in epithelial tumours [31]. Furthermore, MICA protein acts as a signal during the early immune response against infection and a lower level of this protein is thought to decrease the ability to alert the immune system of HPV infection, thus increasing the risk of tumour development [10]. Six distinct alleles of a (GCT)n triplet repeat polymorphism have been described within *MICA*, including the A5.1 allele, correlated with rs2516448, characterized by an additional insertion of guanine after the second triplet, which creates a frame shift mutation resulting in a *MICA* truncated protein lacking part of the transmembrane domain. Rs2516448 is in perfect LD (r^2^ = 1.0) with A5.1 in Europeans, a frameshift mutation of MICA [10]. *MICA–A5*.*1* variant has been implicated in many immune-related diseases, including CC [32], thus supporting its role in immune response and tumour development. Interestingly, a 1.8 fold increased risk of developing CC was reported for *MICA*-*A5*.*1* homozygote carriers, whereas *MICA*-*A5* homozygotes had nearly threefold protection against CC [32].

In the present analysis, we decided to report the original p-values, without correction for multiple testing. However, the success in replicating the results of Chen et al, 2013 [10] in different populations and the results from biological studies suggests a real association of this SNP with CC. Conversely, within the same MHC-CHR6 region and contrary to the findings reported by Chen et al, 2013 [10], we did not observe a statistically significant association for a proxy of rs3117027, i.e., rs3129269. This may due to population differences related to the alleles in the present study population and in the Swedish population, particularly as this SNP is located within the complex LD structure of the MHC region.

Shi et al [12] reported significant associations for SNPs rs8067378 in CHR17 and rs13117307 in the EXOC1 region. In our study, however the associations were not statistically significant. Similarly, the association of SNP rs2665390 in *TIPARP* with CC, which has been previously associated with ovarian and breast cancer [33], did not reach a level of significance in our study. Carriers of TC alleles showed an approximately 40% increased risk of CC as compared to TT carriers, whereas in [33] CC carriers had a 25% increased risk of ovarian or breast cancer as compared to TT carriers.

We were also able to assess the association between SNPs and HPV-positivity among control women. None of the SNPs that were significantly associated with CC were also associated with HR-HPV positivity among controls, with the possible exception of rs13117307 in the EXOC1 region for which, however, the direction of the association with HPV positivity among TT carriers was opposite to that with CC. This discrepancy is not, however, implausible as the genetic determinants of susceptibility and persistence of HPV infection may well differ from those of malignant transformation. Indirect support for the distinction between the potential for persistence and neoplastic transformation comes from studies of the viral genome which showed that evolutionary distinct HPV types, that differ from each other by at least 10% in the DNA sequence of the L1 ORF, have varying probabilities of persistence and, given persistence, different probabilities of neoplastic progression [5]. Conversely, we cannot exclude that the inverse association of borderline statistical significance with HPV-positivity observed for the AA allele of TERT rs2736100 could be a spurious result.

The present study has strengths and weaknesses. The joint assessment of the genetic determinants of HPV persistence and malignant transformation is a major asset as is the application of the same epidemiological and testing protocols (for both HPV and SNPs) to different populations. Weaknesses of the present study include, however, the restriction to a selection of *a priori* interesting SNPs, the relatively low statistical power for the study of polymorphisms that vary in both frequency and LD structure across populations, and the impossibility to asses jointly the influence of genetic characteristics of the host and of the virus, e.g., types and variants.

In summary, this study confirms the association with the rs2516448 variant suggesting that the MHC region is involved in the CC process. The underlying mechanisms that mediate changes in CC disease remain however to be elucidated and further GWAS-based investigations are required, in particular to demonstrate the stage of the carcinogenic progress at which these genes may be involved.
