# Correction: Immuno-related polymorphisms and cervical cancer risk: The IARC multicentric case-control study

**DOI:** 10.1371/journal.pone.0181285

**Published:** 2017-07-07

**Authors:** James McKay, Vanessa Tenet, Silvia Franceschi, Amélie Chabrier, Tarik Gheit, Valérie Gaborieau, Sandrine McKay-Chopin, Patrice H. Avogbe, Massimo Tommasino, Michelle Ainouze, Uzma Hasan, Salvatore Vaccarella

The seventh author’s name is incorrect. The correct name is: Sandrine McKay-Chopin.
